# Acoustic spectra processing for the determination of cavitation threshold in a high-frequency sonoreactor in water and PEG mixtures from 1 to 54 mPa.s^☆^

**DOI:** 10.1016/j.ultsonch.2025.107388

**Published:** 2025-05-17

**Authors:** V. Avramovic, L. Hallez, C. Inserra, J-Y Hihn

**Affiliations:** a Université Marie et Louis Pasteur, CNRS, Institut UTINAM UMR 6213, F-25000 Besançon, France; b Univ Lyon, Université Claude Bernard Lyon 1, Centre Léon Bérard, INSERM, UMR 1032, LabTAU, F-69003 Lyon, France

**Keywords:** Hydrophone, Dosimetry, Acoustic cavitation, Stable cavitation, Transient cavitation, Viscous media

## Abstract

Ultrasound-induced cavitation, especially its stable and inertial regimes, plays a critical role in sonochemical processes. Thus, finding the power thresholds differentiating the appearance of stable cavitation and then transient cavitation, is essential for sonoreactor characterization since this knowledge allows the relevant choice of operating parameters leading to the expected sonochemical effects. However, this concept is difficult to grasp and often applied with complex techniques difficult to implement and highly “system dependent”. The study of sonochemical activity and efficiency in a high frequency reactor (575 kHz) provides an opportunity to take an interest in the step-by-step implementation of a hydrophone measurement, from selection of the device to recording procedures, and especially signal processing with selection and extraction of the two most relevant probes: 3F_0_/2 magnitudes useful for detecting stable cavitation and cumulative integration of broadband noise and its interpretation (slope discontinuity) to get an indication of transient cavitation appearance. An original result of this work is that this technique appears to be much more reliable and sensitive than chemical techniques when media are very viscous, remaining relevant beyond the limits of sonochemiluminescence and dosimetry techniques. This is interesting because it makes it possible to link evolution of the change thresholds of cavitation state (from absence of cavitation to stable cavitation, then transient cavitation) with an increase in viscosity. As expected, these thresholds increase, from 0.25 W- stable cavitation − and 2 W – inertial cavitation − in water up to 6 W for stable cavitation and an absence of inertial cavitation at 54 mPa.s, as it is necessary to apply more power to achieve cavitation in high viscous media. This range of magnitude of viscosity used in this study is relevant for specific applications, such as ultrasonic cleaning lines involving cleaning solutions and all scientific issues dealing with deep eutectic solvants (DES) for electropolishing or leaching processes in metal recovery. A constant monitoring by measuring regularly these parameters ensures that cleaning or treatment lines are running at constant efficiency and helps to identify critical breakdowns. Finally, what is remarkable is that the stable cavitation threshold seems to be directly proportional to the acoustic field (linear dependence of the stable cavitation threshold) if the latter is expressed in pressure, providing new challenges for acoustic fields modelling.

## 1 Introduction

Since its emergence in the middle of the 20th century, particularly for industrial cleaning [1], ultrasonic applications have multiplied as the effects are useful for chemical processes such as polymerization [2,3]. After industry, medicine and cosmetics have appropriated the cavitational properties of ultrasound in liquid media. The acoustic power applied in a reactor filled with water medium is well known to create bubbles that oscillate and that collapse violently enough to lead to interesting chemical effects by applying more power, known as the sonochemistry effect. Increasing the efficiency of this effect is currently sought by different means, such as the control of the applied power or by varying the driving frequency, amongst other more sophisticated techniques [4].

Triggering cavitation in a medium requires the increase of the transmitted acoustic power in order to exceed some cavitation threshold. Above this threshold, cavitation bubbles expand from medium nuclei and oscillate in the regimes of stable and/or inertial cavitation depending on the surrounding conditions (mostly the applied acoustic pressure, the environmental parameters and the ultrasound sequence). It is worth noting that, once the microbubbles are generated, both cavitation regimes can coexist in the sonicated medium [5]. Several parameters must be taken into account, such as volume, temperature, position of the elements in a reactor, and formation of free radicals coming from the solution. As a consequence, predicting the threshold of inertial cavitation remains difficult. Several methods already exist to determine cavitation:

– the use of a sheet of aluminum foil which can disrupt propagation of ultrasound in the solution,
– calorimetry which takes a long time to implement and requires an increase in temperature, which can prove critical on living cells, for example [6].
– dosimetry (with its range of methods) is a good candidate but is reserved for experienced personnel [7].

Moreover, since pioneer works of E.A. Neppiras et al [8], it appears interesting to use hydrophones to detect and monitoring cavitation from acoustic spectra recording. More recently, several improvements have been proposed to this seminal work. B. Zeqiri et al for example have designed a novel acoustic sensor for monitoring bubbles exposed to an acoustic field [9], and show that their prototype works in broad range of frequencies, from 20 kHz to 20 MHz [10]. Firstly developed for melted metals [11,12], a cavitometer was used by I. Tzanakis et al for various liquids such as water, glycerin and ethanol. A significant amount of work has been devoted to the study of the triggering of the various cavitation regimes, with attention given to the intensity distribution in the reactors (distance to horn) [13]. In particular, they have shown that it is possible to detect transition between stable and inertial cavitation, and that it might be possible to quantify the cavitation activity. Nevertheless, only low frequency emission (20 kHz) have been studied, and no systematic treatment of the spectra have been proposed.

The present study proposes the use of a hydrophone (marketed product) to detect cavitation autonomously. Use of the signal from the hydrophone can be carried out by computer processing within everyone's reach and of reasonable cost [[14], [15], [16]]. This is subsequently applied to the analysis of several solutions of different viscosities in order to determine their cavitation thresholds because as mobility is reduced, cavitation is more difficult to obtain [17].

In this objective, a step-by-step implementation of a hydrophone measurement has been undertaken with a progressive and detailed methodology, from selection of the device to recording procedures, and especially signal processing with selection and extraction of the two most relevant probes on hydrophone signal. A particular care was given to the calibration and transmitted power measurements, because this point is sensitive to the viscosity (changes in absorption and reflections), which lead to adapt the electrical power in function of the system impedance. It is important to note that this protocol was first tested at 20 kHz, but it was less interesting because cavitation thresholds of inertial and stable cavitation were reached simultaneously for low intensities. Therefore, the analyzed signals were only taken at high frequency to highlight more interesting transitions, allowing a deeper methodological description.

Then, several treatments and methodologies for signal processing have been implemented and their relevance evaluated. Although these methodologies are available in the literature or subject to standards, they are not always compared and their application is often linked to a specific scientific community. This work is therefore also the opportunity to evaluate this type of treatment when applied to a sonochemistry problem, with a counter-check in the form of dosimetry and sonochemiluminescence measurements for cavitation thresholds determination [18,19].

## 2 Experimental details

### 2.1 Experimental set-up

**Fig. 1:**
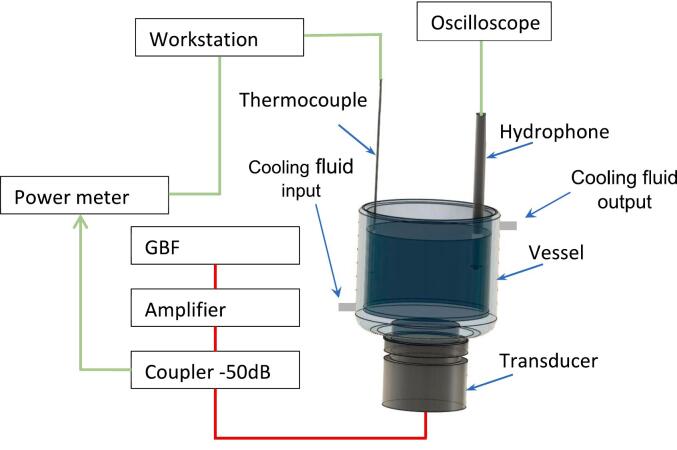
Schematic of the experimental set-up.

Experiments were carried out in a 150 mL glass (50 mm inner diameter and corresponding liquid height 72 mm) with colling jacket buckled on a E/805/T multifrequency transducer designed by Meinhardt® (Leipzig, Germany), working at 575 kHz – 858 kHz and 1138 kHz. It generates a continuous sinusoidal wave tuned by the voltage output of a function generator (Agilent® 33500B series: DC up to 20 MHz), and a constant gain amplifier (Research® 150A 100B amplifier: 10 kHz up to 100 MHz, 150 W) (Fig. 1). The electrical power emitted by and returned to the amplifier is measured through a bi-directional coupler (−50 dB) with a milliwatt-meter (Rohde&Schwarz® NRVD: from 9 kHz to 2 GHz). It identifies the effective power absorbed by the transducer. Local acoustic pressure was measured with a hydrophone connected to an oscilloscope (Keysight® Infiniivision 4000series: 1.5 GHz, 5GSa/s) that treats data to show FFT (Fast Fourier Transform). Temperature was monitored with a thermocouple associated with a regulator to perform all experiments at room temperature (around 21–23 °C) under atmospheric pressure. Note that the hydrophone was located at the same position in all experiments i.e. at 5 mm from external wall and 10 mm depth, so that the wave alteration will be restricted to the same range of magnitude and therefore neglected.

### 2.2 Properties of irradiated liquids

Viscous media were formulated from ultra-pure water (0.055µS) mixed with various amounts of polyethylene glycol 6000 PEG as shown in Table 1 as representative of different ionic liquids chosen in reference to a former work [20,21]. All samples were saturated by air bubbling for 10 min. For each concentration of PEG into water, viscosity was measured by rheometer Kinexus MalVern®, and thermal capacitance was characterized by differential scanning calorimetry METTLER® (Table 1).

**Table 1:** Viscosity and heat capacity of medium.

PEG concentration (mg.g^−1^)	Viscosity at 25 °C (mPa.s)	Heat Capacity (J.Kg^−1^.K^−1^)
0.0 (pure water)	1	4190
20	2.4	4214
40	3.2	4227
75	4	4249
100	5	4259
147	6.1	4277
210	12	4378
253	15.1	4432
325	32	4722
390	54	5100

### 2.3 Sonochemical activity evaluation

Dosimetry was used to detect peroxide production as witness of the chemical cavitation activity. Samples of 1 mL of mixture taken in the sonoreactor were mixed with a solution of ammonium heptamolybdate (10–3 mol.L-1) and potassium iodine (10–2 mol.L-1), while absorbance was measured at 350 nm using a UViline® spectrophotometer (type 9400C). [18]. Sonochemical luminescence was measured in NaOH (10^-1^ M) and Luminol (2 mM) mixtures with various PEG concentrations saturated in air. The set-up was placed in a black box. Pictures were taken with a Canon® EOS 550D camera, with F4.0. diaphragm aperture, while the shutter remained open for 25 s at ISO 6400 sensitivity simultaneously to photon counting with an optical fiber connected to a Hamamatsu® H10721-210 photomultiplier.

### 2.4 Hydrophone selection

**Fig. 2:**
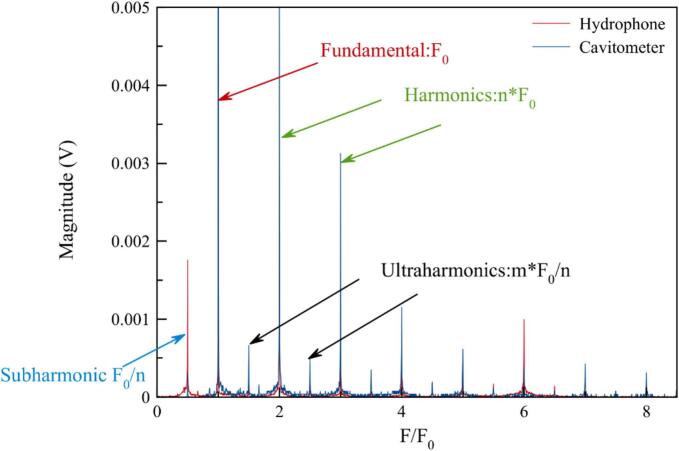
Acoustic spectra of two hydrophones in the same reactor at 575 kHz in air saturated water and in an inertial cavitation mode.

Among the various hydrophones at our disposal in the lab (Submarine hydrophone Brüel&Kjaer 8103, Cavitometer ICA-3MHF1 Belarussian State University of Informatics and Radioelectronics, IMASONIC T040-0101, homemade hydrophones), a selection was made to choose the most sensitive to our driving frequency [SM1]. Even if Cavitometer ICA-3MHF1 was designed for low frequencies, it shows a high sensitivity to broad band noise and peak definition (Fig. 2), especially at 3F0/2 in cavitation conditions, which is an important criterion in our study. A comparison between two very different hydrophones (Brüel&Kjaer 8103 and ICA-3MHF1) is shown as an example in Fig. 2. This is an opportunity to identify fundamental frequency (f_0_), harmonics (n*f_0_), subharmonics (f_0_/n), and ultraharmonics (m*f_0_/n).

### 2.5 Transmitted power calibration and control

The calorimetry method is used to determine acoustic wave power transmitted to the sonoreactor reassociated with the functional media for different viscosity and excitation levels, with a thermocouple placed along the reactor wall to be outside of the acoustic field [6]. During the calorimetric experiments, cooling fluid circulation was stopped, initial temperature was chosen close to room temperature and the duration was limited to the first seconds (duration is dependent of the range of power). In those conditions, the temperature increase was linear, confirming adiabatic conditions. Transducer yield is defined by the acoustic on electrical power ratio, measured vs. liquid viscosity. These transducer yields are recorded either by incrementing output voltages strictly set at a given value, by 10 mV steps up to 150 mV, or in a regulation mode based on a feedback loop associated with the power meter with a custom-made LabView program (see the scheme in Fig. 1) [22]. For this purpose, a workstation was added to regulate generator voltage, enabling the electrical efficient power absorbed by the transducers to remain constant. This allows a more stable acoustic transmission performance in this servo mode (+/- 0.5 W.).

**Fig. 3:**
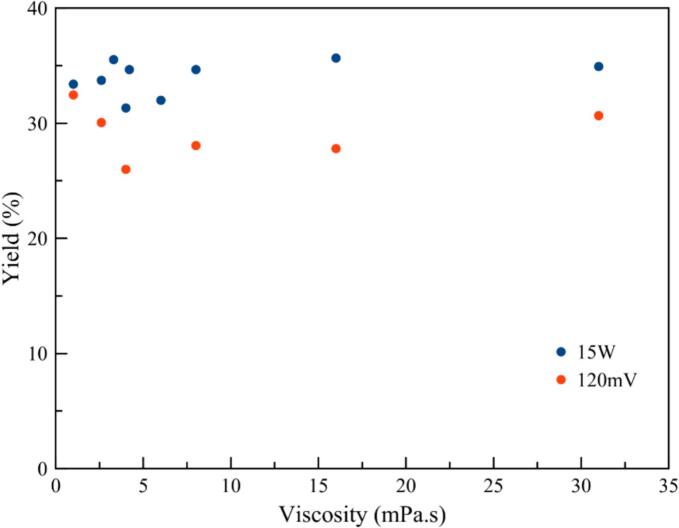
Transducer efficiency as a function of viscosity: excitation voltage set at 120 mV at the generator output (blue dots) or with a regulated power of 15 W (red dots).

**Fig. 4:**
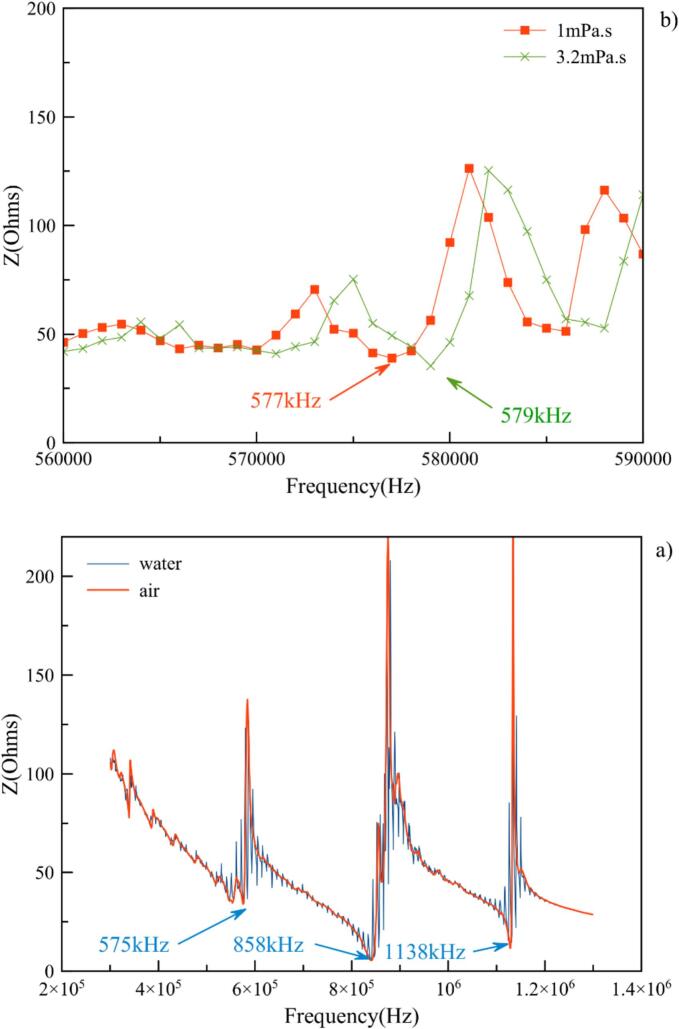
(a) Real part of impedance Z as a function of transducer frequency for pure water (1 mPa.s^−1^) and air (17.2 µPa.s^−1^) − (b) Real part of impedance Z as a function of transducer frequency for pure water (1 mPa.s^−1^) and PEG solution (3.2 mPa.s^−1^). Zoom in on the area of interest around 575 kHz.

Plotting the results of transducer yields measured both at a given output voltage of 120 mV (blue dots in Fig. 3) and at a regulated transmitted power of 15 W (red dots in Fig. 4), a greater dependence on viscosity at a fixed voltage can be observed, with a minimum (down to 23 %) of around 4 mPa.s. On the contrary, with the feedback loop on transmitted power, the dependence is less visible.

This decrease in transducer yield of around a few mPa.s can be attributed to an impedance mismatch, which results in a deviation from optimum transmission at a given frequency.

This phenomenon is presented in Fig. 4a, which shows the frequency-dependent measurements of the real part of the electrical impedance of the transducer using a RLC bridge “FI8110G” in air and in water. The three resonant frequencies can be clearly identified at 575 kHz, 858 kHz, and 1138 kHz, without load (red curve) and in water (blue curve). It is interesting to note that anti-resonance peaks, as well as resonance minima, are better defined in air than in water.

Comparing two solutions with different viscosities (1.0 mPa.s^−1^ and 3.2 mPa.s^−1^) around the area of interest of the impedance diagram (around 575 kHz), a shift in resonance is observed from 577 to 579 kHz (Fig. 4b). Indeed, this slight frequency shift can induce a greater variation for impedance at the defined working frequency, leading to noticeable yield loss. To overcome this problem, ultrasonic equipment manufacturers frequently tune the driven resonant frequency to minimize the electrical power reflected to the generating system. This is problematic for comparing electrical and transmitted power at a given frequency, particularly for very high viscosities.

**Fig. 5:**
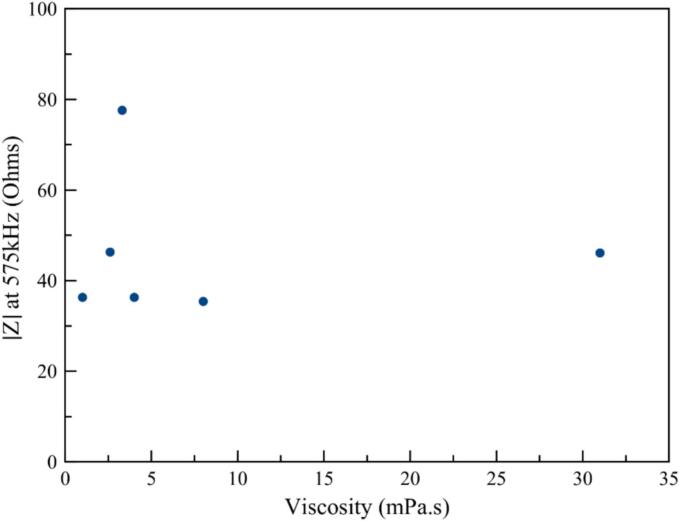
Transducer electrical impedance at 575 kHz as a function of medium viscosity.

An illustration is obtained by plotting the real part of transducer impedance as a function of viscosity for 575 kHz frequency (without tuning) in Fig. 5, which confirms the differences previously observed for the full range of viscosities. The large variations observed, especially around 4 mPa.s, are consistent with the decrease in yield detected in Fig. 3, since the higher the electrical impedance of the system, the lower the electroacoustic conversion efficiency.

**Fig. 6:**
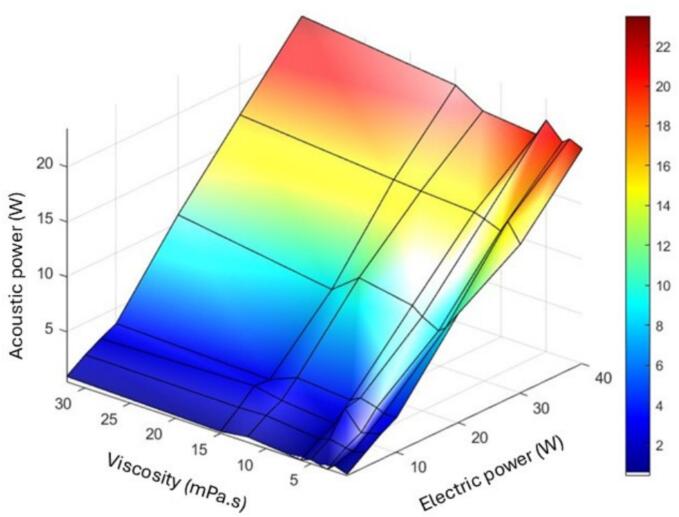
Acoustic powers vs. viscosity as a function of electrical power input in the case of a control loop.

Finally, to broaden the view by grouping together in one figure the effect of electrical power and the effect of viscosity on acoustic power, it can be observed that the variations induced by viscosity is corrected by our control loop. All results are grouped in Fig. 6, which can be used as an abacus for direct acoustic power prediction.

This confirms that using a regulation mode based on a feedback loop associated with the power meter warrants a quite constant acoustic power for each viscosity.

## 3 Cavitation parameter extraction from acoustic spectra recorded in water – Data correction and spectrum analysis in water

This paragraph is concerned with the recording of acoustic spectra and their processing in order to get information about cavitation activity. Acoustic spectra are recorded with Cavitometer ICA-3MHF1 connected to the oscilloscope and converted into an analog electrical signal. Multiple measurements are taken by varying acoustic power. The following settings are chosen: Option: FFT, Window: Hanning, Average: 32, Scale: 100 µV, Offset: 300,000 µV, Coupling: DC, Impedance: 1 MΩ, Sample rate: 31.3 MHz, Frequency range: 0–1.3 MHz. The FFT spectra, quoted in tension gain expressed in volts or dB, are exported, processed, and analyzed using spreadsheet software. The scale of 100 µV do not allow the record the highest peaks values, but increase the resolution in the spectra bottom without losing relevant information as 3F_0_/2. This has the consequence of minimizing the weight of the peaks corresponding to the discrete frequencies (fundamental, harmonics or ultraharmonics) that emerge from the broadband noise. But it has been checked that, due to the high quality factor and sharpness of the emerging peaks, neglecting their weight during full spectra integration from F/F0 = 0 to F/F0 = 2.5 led to a higher sensitivity in broadband noise quantification. An example of evaluation is presented in SM2.

**Fig. 7:**
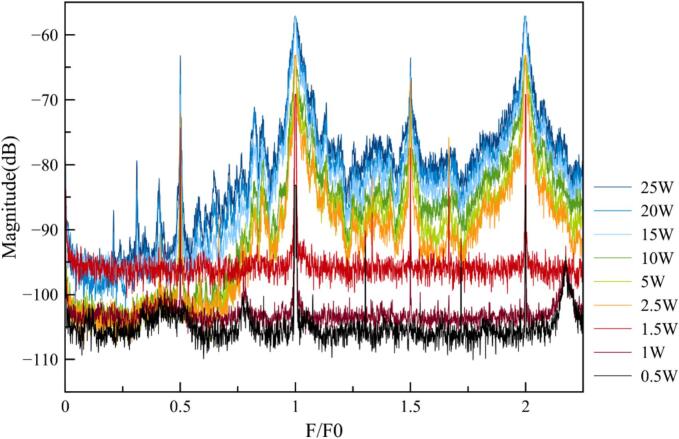
Raw spectra recorded at various transmitted powers.

Looking at the raw data (Fig. 7), it is obvious that processing is necessary as it is difficult to directly link the effect of power increase to the cavitation parameters. First, data collection is not straightforward and may vary depending on daily operating conditions, especially the baseline which should be exactly the same outside of hydrophone bandwidth. Second, it is not obvious from the literature to identify the relevant parameter which will make it possible to link cavitation activity quantification to acoustic spectra treatment. Several protocols still exist and will be evaluated below. Nevertheless, even raw data provide interesting insights into cavitation behavior. For example, the peaks occurring at the transducer frequency (F_0_, fundamental), as well as harmonics (n*F_0_), are very thin for low power inputs but become larger for higher ones. This observation may be linked to several phenomena, such as dispersion of bubble sizes around their initial ones. This is still visible, but to a lesser degree for ultraharmonics (m*f_0_/n), which correspond more to bubble motion in the acoustic field [23,24].

### 3.1 Raw spectra correction

**Fig. 8:**
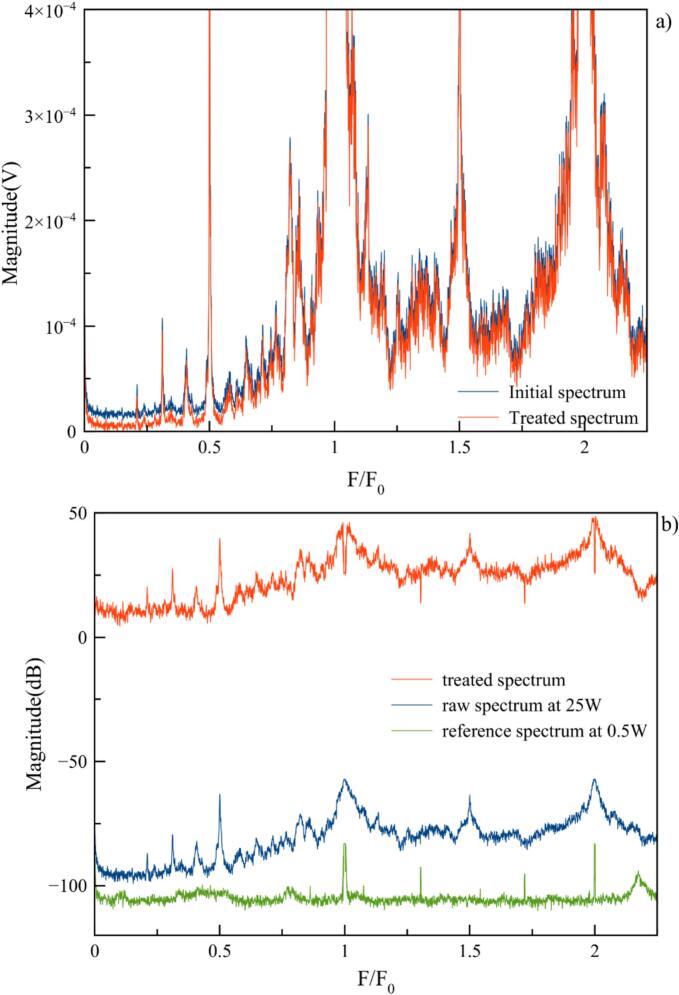
A) raw spectrum in volts recorded at 25 W (in blue) is shifted down so that its minimum value is equal to zero Volts – treated spectrum in red b) After conversion into dB, raw spectrum in blue is corrected from 0.5 W reference spectrum in green – treated spectrum in red.

Therefore, to improve experimental repeatability and to free data from system variations, the possibility of a pretreatment was investigated. Two possibilities were tested: i) prior to conversion into dB, all spectra are shifted down so that their minimum value is equal to zero Volts (Fig. 8a) ii) after conversion into dB, a reference spectrum corresponding to the environmental noise (recorded at 0.5 W, i.e. in absence of cavitation) is systematically subtracted as illustrated in Fig. 8b. While it is obvious that curves evolve in a similar manner, regardless of the processing method, the best results were obtained by environmental noise substraction, as commonly used in medical research [16]. This will be applied for all further spectra.

It could be interesting to note that the low frequency part of the spectrum (below 100 kHz) appears flat on Fig. 8 due to the low sensitivity of Cavitometer ICA-3MHF1 in this zone. As explained in the hydrophone selection section, equipment performances are limited to an optimum zone.

### 3.2 Spectra analysis – Identification of cavitation probes

**Fig. 9:**
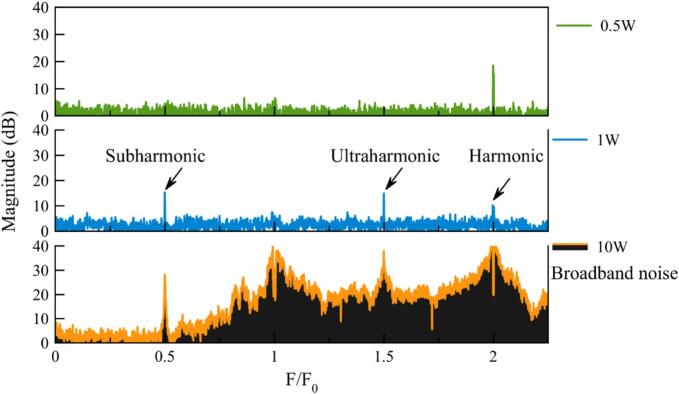
A zoomed-in view of the spectrum is presented, corresponding to three power levels associated with three cavitation states in pure water. Before displaying the results, the noise spectrum (measured without cavitation) was subtracted in dB.

To identify a methodology for detecting cavitation through spectral analysis, data were extracted or calculated from spectrum measurements. Fig. 9 shows the evolution of the acoustic spectra as a function of the transmitted power after spectra correction. Three typical states can be observed: below cavitation threshold (0.5 W) with no peaks, as the fundamental frequency was removed by correction – at 1 W with the appearance of subharmonic peaks – at 10 W with the presence of broadband noise.

Fig. 9 presents spectra obtained in water at different acoustic power levels. At 0.5 W, no cavitation is observed. At 1 W, stable cavitation is identified by the presence of ultra-harmonics at 3F_0_/2 [1], corroborated by visual observation of bubble layers through the glass reactor. At 10 W, transient cavitation is detected, characterized by broadband noise [2], further confirmed by sonochemical luminescence (SCL) and dosimetry, as detailed in the “Dosimetry” section.

After removing the environmental noise, spectra obtained at various transmitted powers (Fig. 7) were processed to select the best cavitation probe for intensity quantification [SM3]. Several possibilities are available and were implemented. This is sometimes very confusing for sonochemists, as results are strongly dependent on the set-up (hydrophone position, etc.), on the range of magnitude of the powers used, and finally on the original scientific community conducting the test (medicine, mechanics, etc.).

**Fig. 10:**
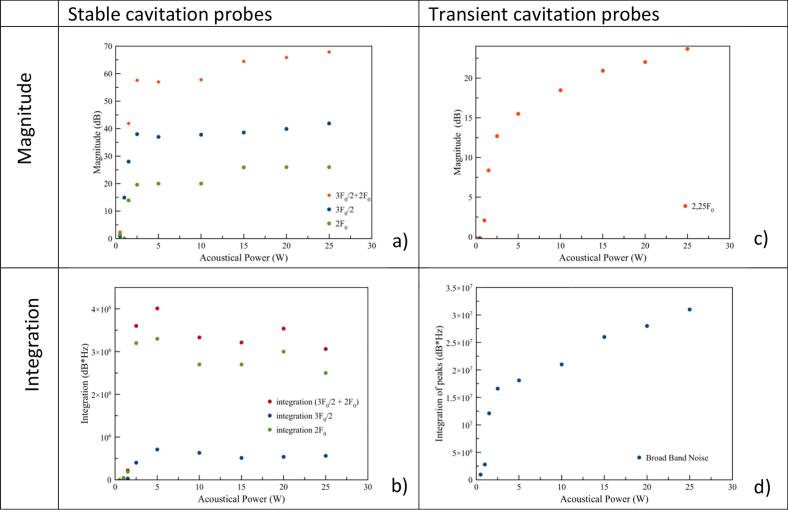
Stable cavitation probe comparison: Harmonic 2F_0_, ultraharmonic 3F_0_/2, and their sum, a) Magnitudes b) Integrations – stable probe comparison: c) Magnitude of 2.25 F_0_ d) Broadband noise integration.

Among those protocols used to quantify stable cavitation, magnitudes (Fig. 10a) and integrations (Fig. 10b), as well as their sum, are recorded at 3F_0_/2 [15] or at 2F_0,_ and plotted vs acoustic power. On the other hand, for transient cavitation, measurement of gains in tension at 2.25 F_0_ recommended by the norm [IEC TS 63001] is plotted (Fig. 10c), as well as broadband noise integration (full spectra without peaks) [5] (Fig. 10d).

Concerning stable cavitation, magnitudes start to increase above a threshold at very low acoustic powers for all cases (3F_0_/2 and 2F_0_), and rise rapidly to reach an upper level, not totally stable (Fig. 10a). It is important to notice that all values have been corrected from environmental noise, which explains the low magnitudes observed for 2F_0_, and to keep in mind that acquisition scale was limited to 100 µV, which can removed the highest peaks values. Integrations were made on the complete axis range i.e. between F/F_0_ = 0 to F/F_0_ = 2,5, but unfortunately, they do not provide additional information, presenting more variations in upper levels (Fig. 10b). Finally it is the ultraharmonic magnitudes recorded at 3F_0_/2 which present the highest sensitivity. As this is the simplest post-treatment, it will be kept for further testing. For transient cavitation probes, both 2.25F_0_ magnitude (Fig. 10c) and broadband noise integration (Fig. 10d), extracted from spectra, show an increase before a slope discontinuity, indicating the inertial cavitation threshold according to ISO [IEC TS 63001]. Nevertheless, since slope discontinuity is more pronounced in the case of broadband noise integration, this parameter has been kept for further post-treatment. Following the magnitude at 2.25F_0_ is acceptable, but in our case its variation is disturbed by the enlargement of the 2F_0_ peak as shown in Fig. 7.

### 3.3 Threshold detection

**Fig. 11:**
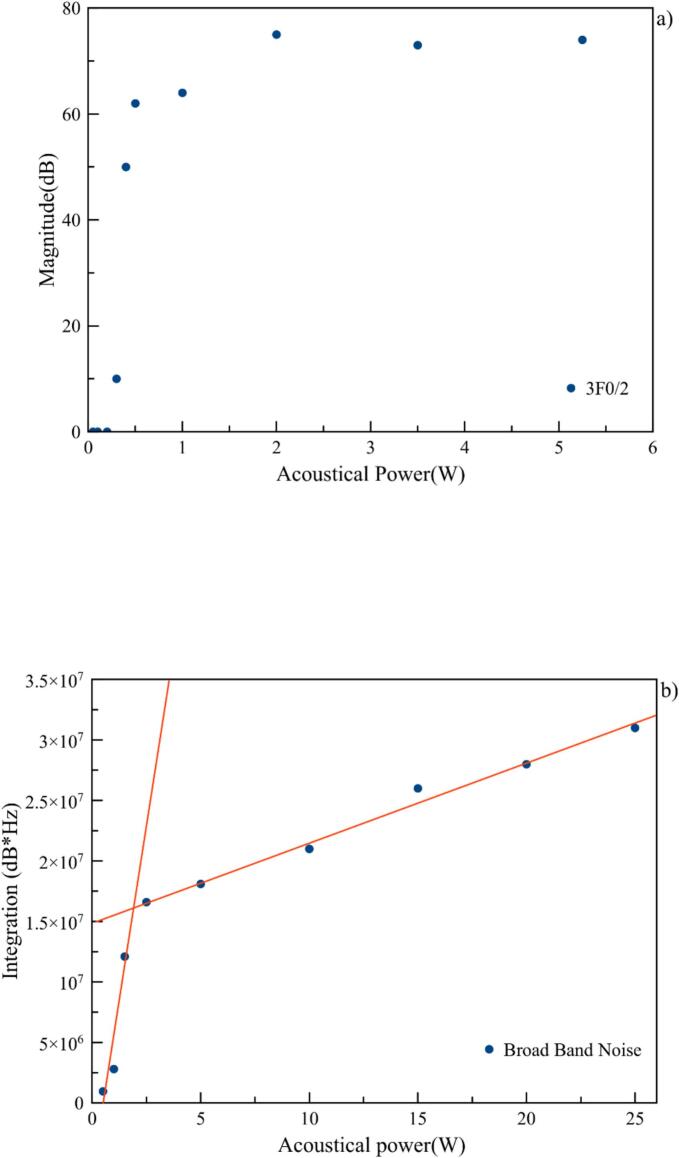
A) magnitude of ultraharmonic 3f_0_/2, b) Broadband noise integration.

The aim is to implement these techniques to find the different cavitation thresholds. For stable cavitation, measurements of magnitude at 3F_0_/2 were repeated with more points in the low power range. This is an opportunity to observe the dependence of absolute values on the system, as the range of magnitude of the recorded values is far higher at equivalent acoustic power input (around 80 dB instead of 40 dB at 5 W). Nevertheless, the global pattern remains the same, and the threshold is determined at a signal appearance, i.e. 0.25 W (Fig. 11a). Concerning the transition between stable and inertial cavitation, ICE standard recommendations can be applied, and the threshold is obtained at the slope discontinuity materialized by the intersection at around 2 W (Fig. 11b).

**Fig. 12:**
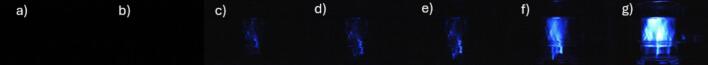
Pictures of the sonoreactor with luminol for various transmitted powers a) 0.6 W, b) 1.8 W, c) 2 W, d) 2.5 W, e) 3 W, f) 5 W, g) 10 W.

As the nature of cavitation can also be detected by chemical methods, sonochemiluminescence as well as chemical dosimetry were implemented to confirm the previous results. Fig. 12 shows pictures captured for increasing transmitted powers, ranging from 0.6 W to 10 W. Sonochemiluminescence appears as from 2 W and is brighter as power increases, while the active zone becomes larger, spreading throughout the reactor volume.

**Fig. 13:**
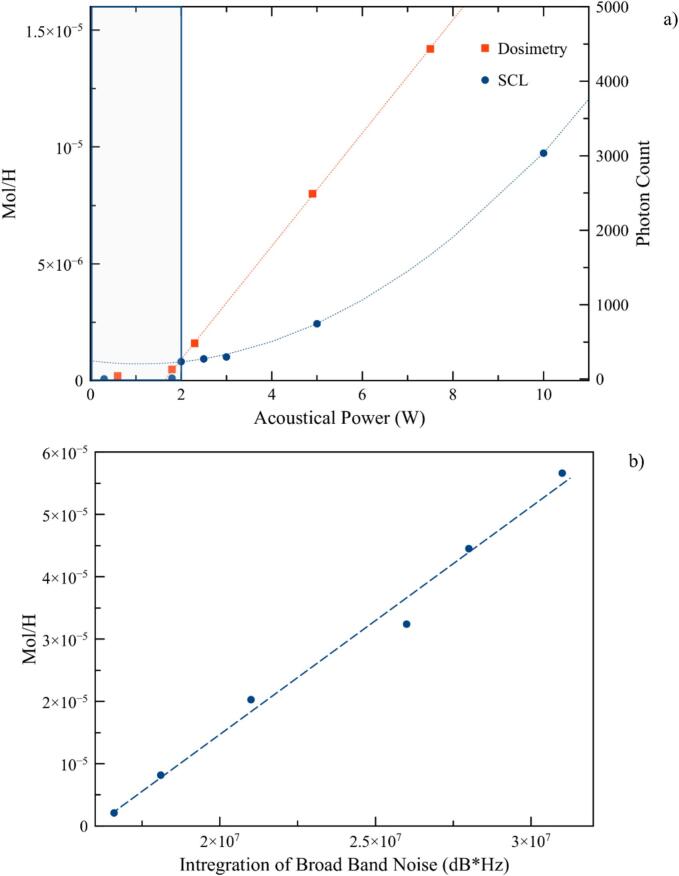
A) h_2_O_2_ production rate mol.h^−1^ and photon count in 5 ms vs. transmitted power W b) H_2_O_2_ production rate mol.h^−1^ vs. Broadband noise integration.

**Fig. 14:**
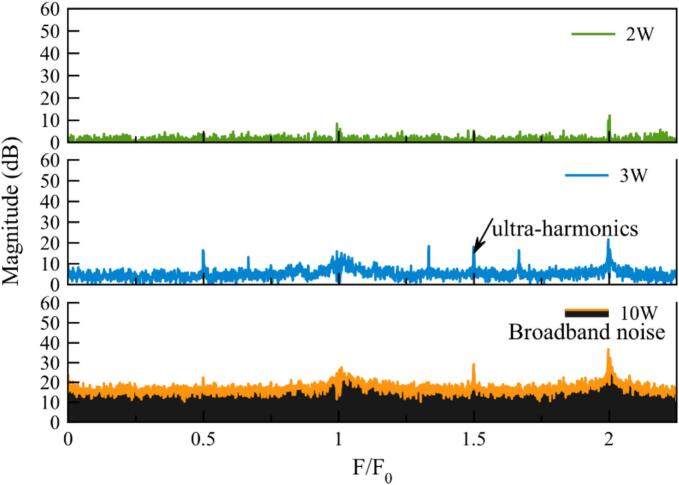
A selection of post-treated spectra is shown, corresponding to three power levels associated with three cavitation states in 2.4 mPa.s (20 mg/L in PEG). Before displaying the results, the noise spectrum (measured without cavitation) was subtracted in dB.

Since quantification by this method is not easy, an optic fiber was placed close to the luminous zones and connected to a photomultiplier to count the photons for a period of 5 ms. Values obtained are plotted vs. transmitted power on the same graph as the H_2_O_2_ production rates (Fig. 13a). Both chemical methods, indicating hydroxide generation typical of transient cavitation, present a threshold between 1.8 and 2 W. [SM4]Fig. 14..

This is in perfect agreement with the measurements obtained by hydrophone, which give an inertial cavitation threshold determined from the wide-band noise as early as 2 W. The lower power values, even if detected by the magnitude of 3F_0_/2, correspond well to a stable cavitation, i.e. which has no sonochemical efficiency. Moreover, plotting the H_2_O_2_production rate as a function of broadband noise gives a linear relationship (13b), leading us to think that broadband noise integration may be useful as an indicator of chemical activity. This could be of great help in many situations by avoiding use of complex methods or chemicals for this analysis.

All data are available in Supplementary Material [SM5].

## 4 Cavitation parameter extraction from acoustic spectra recorded in water + PEG – Influence of viscosity

**Fig. 15:**
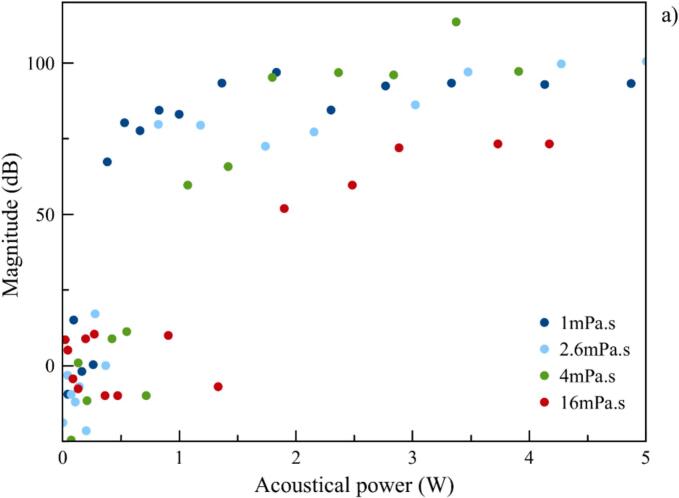
Gain in tension of 3F_0_/2 at various viscosities ranging from 1 to 16 mPa.s.

In this section, acoustic spectra are obtained under the very same conditions as above (same recording conditions, same pretreatment, etc.) in a water + PEG mixture at several PEG concentrations to allow viscosity to vary. An example of a spectrum obtained with a 2.4 mPa.s mixture at three distinct cavitation states (2 W without cavitation – 3 W with stable cavitation – 10 W with transient cavitation) is shown in Fig. 15.

Comparing these spectra with those obtained in water (Fig. 9), it can be noticed that the stable cavitation state spectrum generates more harmonics, illustrating a possible increase in the number of cavitation bubbles as well as changes in cavitation modes. It is important to note that the viscosity influences the speed of the sound [25,26] without it being possible to define the respective contributions of each parameter (such as the fluid density, PEG concentration).

On the contrary, broadband noise in the transient state situation is more constant irrespective of frequency.

Then, magnitudes of 3F_0_/2, as previously selected with the experiments in pure water (indicators of stable cavitation), are plotted vs. acoustic power for the different viscosities (Fig. 15). Results obtained for each viscosity follow the same sigmoidal global pattern, i.e. a weak signal for the lowest powers before a drastic increase as stable cavitation occurs.

**Fig. 16:**
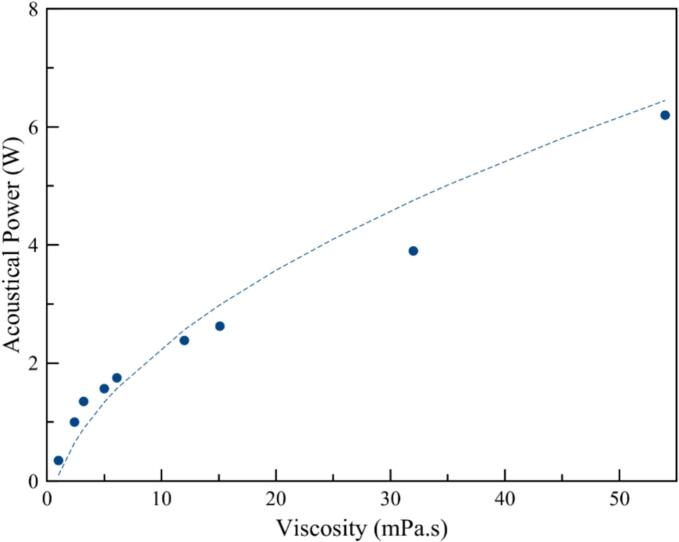
Acoustic power thresholds between absence of cavitation or stable cavitation state as a function of viscosities.

To be able to reach a finer quantification of the acoustic threshold, a new set of experiments were carried out with a direct oscilloscope observation (no data treatment), with a particular care to the power input control. For a given viscosity, minimum powers for peaks apparition at 3F_0_/2 are measured. Looking at dependence of cavitation thresholds as a function of mixture viscosities (Fig. 16), monotonic variation is visible. A monotonic variation is observed and follows a ½ power law. Assuming progressive waves and when expressed in acoustic pressure, the relationship between the threshold pressure and the viscosity becomes linear. This behavior is in good agreement with numerical simulations of the predicted cavitation threshold in viscous media following the model of Holland & Apfel [SM6]. An initial bubble size distribution is chosen in the range 0.05–––3 µm that characterizes the size of the medium nuclei. For the applied driving frequency F_0_ = 575 kHz kHz, the Blake pressure is obtained over the range of nuclei sizes. These pressures are injected in Eq. (11) of the work of Holland & Apfel, which provides an implicit function of the cavitation threshold to be determined. A minimization is performed in order to find this pressure for a given fluid viscosity, whose trend is provided in Figure SM6 as a function of the normalized fluid viscosity.

**Fig. 17:**
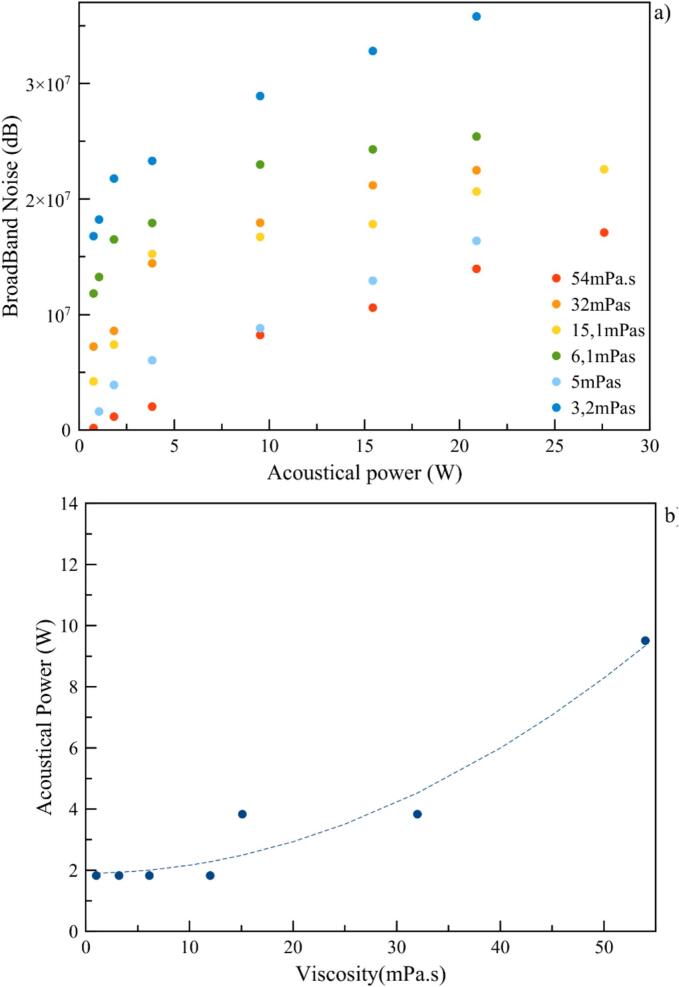
A) magnitude of integration. b) threshold of transient cavitation state in acoustic power depending on viscosities.

Considering the second probe kept for the detection of thresholds between stable and transient cavitation, i.e. broadband noise integrations vs. acoustic powers, a global reduction in absolute values can be seen in Fig. 17a. Indeed, cavitation activity is attenuated in viscous media. This decrease tends to make threshold detection less visible since slope discontinuity (protocol defined by ISO standards) is less marked as viscosity increases. Nevertheless, a global trend can be given by representing the thresholds thus calculated as a function of viscosity, which increase following a polynomial expression of the 2nd order (Fig. 17b).

However, it is no longer possible to relate the results of dosimetry or sonoluminescence as directly as in pure water, with activities that are very clearly slowed down. For example, no SCL activity is observed at 32 mPa. s (no photons detected), and values remain very low at 15 mPa. s (113 photons at 10 W) [SM7-8]. In dosimetry, no results were obtained for viscosities above 5 mPa.s [SM4].

## 5 Conclusion

This study presents a novel, accessible method for detecting and characterizing cavitation phenomena using a commercially available hydrophone and affordable signal processing tools. Indeed, among the several techniques available for sonoreactors characterization, analysis and exploitation of a hydrophone signal in pure water as well as viscous media appears to be an excellent tool for detection of the different cavitation states (stable or transient), as well as their quantification. In particular, it has been demonstrated that **specific spectral features**—notably the 3F_0_/2 ultraharmonic peak and broadband noise—serve as effective, quantifiable markers for stable and inertial cavitation, respectively. These markers correlate closely with independent validation techniques such as sonochemiluminescence and dosimetry. Moreover, it appears to be more reliable in viscous media when these chemical methods have attained their limits. However, the method to implement is not obvious as this subject is mostly treated by different scientific communities, whose objectives and motivations may often differ from those of sonochemists. It was important to cover all the steps, from selection of hydrophones to recording techniques, and especially post-processing on the Fast Fourier Transform signal, with selection and extraction of the two most relevant probes:

– 3F_0_/2 magnitudes which are higher than noise level and useful to detect stable cavitation states. This shows that the higher the viscosity, the greater the acoustic power required to obtain the stable cavitation state. It provides a clear signature of stable cavitation states, as this harmonic is strongly associated with stable bubble oscillations.
– Cumulative integration of broadband noise and its interpretation (slope discontinuity) gives a more sensitive indication of transient cavitation appearance and intensification than the simple magnitude at 2.25F_0_ sometimes proposed. It is well suited to detect transient cavitation states, characterized by high-intensity noise over a wide frequency range. Moreover, it is proportional to the acoustic powers injected into the sonoreactors in the same manner as chemical detection (sonochemiluminescence and dosimetry).

What is particularly attractive with the use of hydrophone measurements is that this technique is much more reliable and sensitive than chemical techniques when media are very viscous, with detections that remain good while those obtained in sonochemiluminescence and dosimetry collapse. This is interesting because it makes it possible to link evolution of the change thresholds of cavitation state (from absence of cavitation to stable cavitation, then transient cavitation) to increase in viscosity. As expected, these thresholds increase, from 0.25 W- stable cavitation − and 2 W – inertial cavitation − in water up to 6 W for stable cavitation and an absence of inertial cavitation at 54 mPa.s, as it is necessary to apply more power to achieve cavitation in high viscous media. This range of magnitude of viscosity used in this study is relevant for specific applications, such as ultrasonic cleaning lines (degreasing, etc.. in surface engineering) involving cleaning solutions and all scientific issues dealing with deep eutectic solvants (DES) for electropolishing or plating, and leaching processes in metal recovery from wastes. A constant monitoring by measuring regularly these parameters ensures that cleaning or treatment lines are running at constant efficiency and helps to identify critical breakdowns.

However, what is remarkable is that the stable cavitation threshold seems to be directly proportional to the acoustic field (linear dependence of the stable cavitation threshold) if the latter is expressed in pressure (not calculated but assumed to be a square root of the acoustic power). This observation is of the highest interest but deserves to be explored in details by calibration of hydrophone data to translate acoustic power into pressure, as well as by modeling the acoustic field itself. Finally, the methodology developed here i.e. hydrophone measurements and data treatment with the extraction of the two most relevant probes may be extended to a wider range of frequency.

## CRediT authorship contribution statement

**V. Avramovic:** Writing – original draft, Methodology, Investigation. **L. Hallez:** Writing – original draft, Investigation, Conceptualization. **C. Inserra:** Investigation, Conceptualization. **J-Y Hihn:** Writing – review & editing, Writing – original draft, Supervision, Methodology, Conceptualization.

## Declaration of competing interest

The authors declare that they have no known competing financial interests or personal relationships that could have appeared to influence the work reported in this paper.
