# Supplementary material for: Acoustic spectra processing for the determination of cavitation threshold in a high-frequency sonoreactor in water and PEG mixtures from 1 to 54 mPa.s
Source: Ultrason Sonochem. 2025 May 17;119:107388. doi: 10.1016/j.ultsonch.2025.107388 (PMC12149647; doi:10.1016/j.ultsonch.2025.107388)
Supplement: Supplementary Data 1 [file mmc1.docx]

SUPPLEMENTARY MATERIAL :


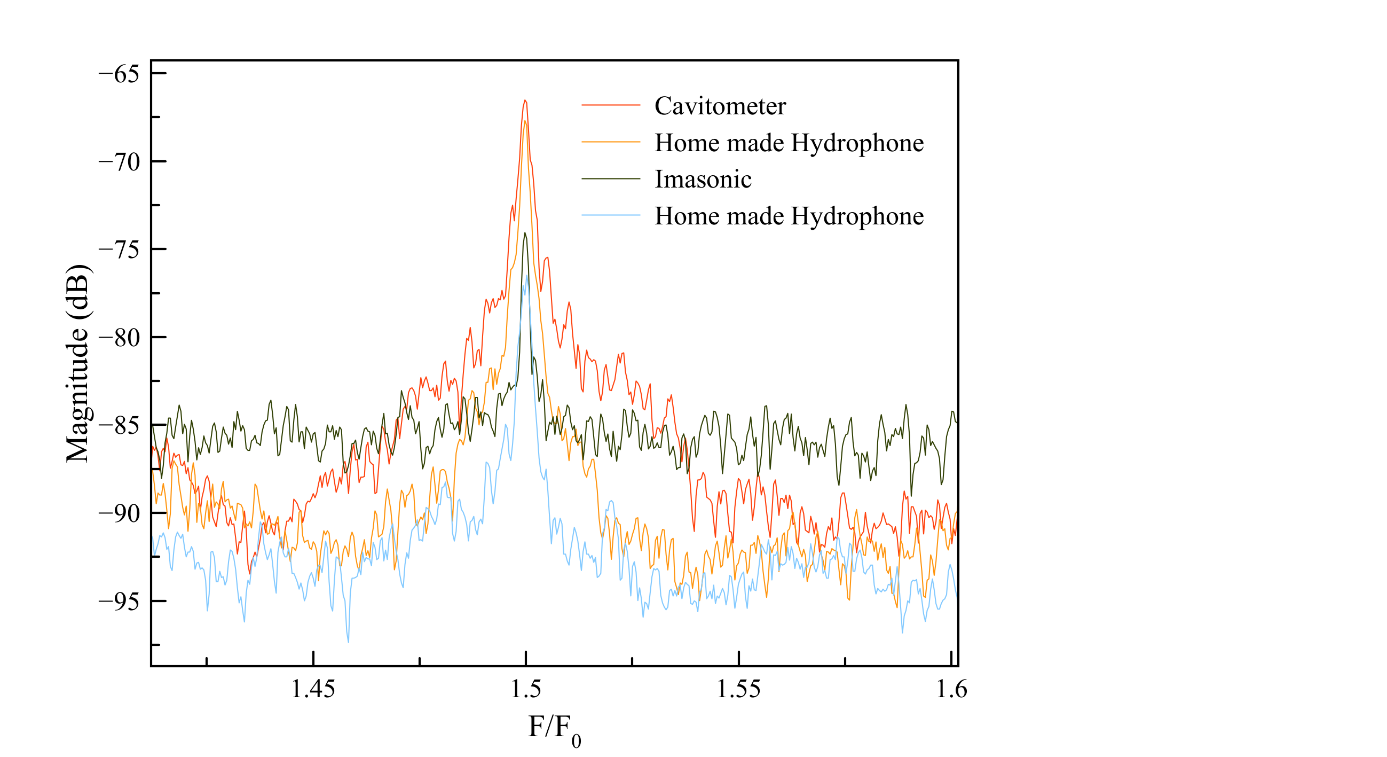


[SM1]: Spectra of several hydrophones into the same reactor at 575kHz and at transient cavitation in water medium.


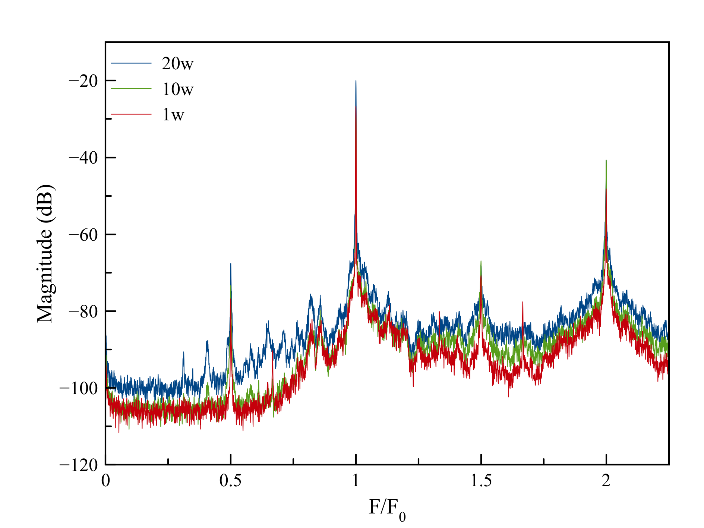

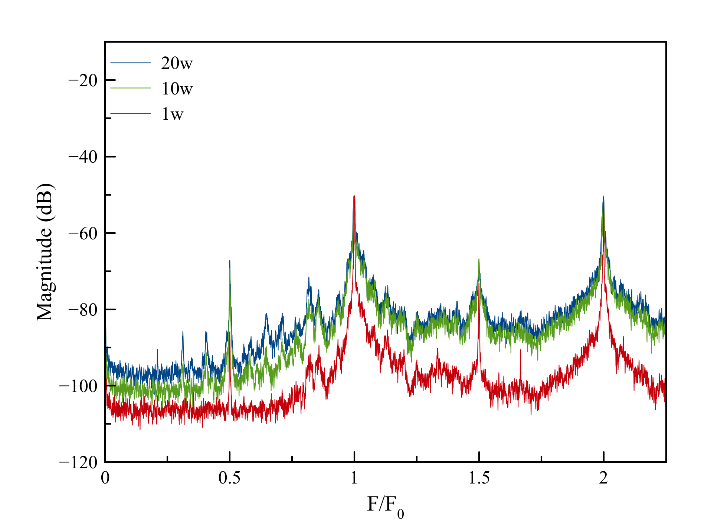
**Raw spectra (recording scale 200µV) Restricted spectra (recording scale 100µV)**

*The FFT displays are quoted in tension gain expressed in dB for different transmitted powers*

*
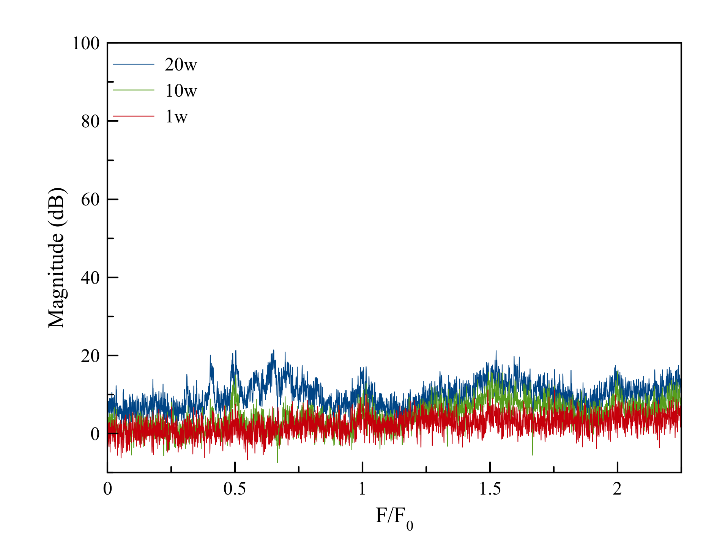

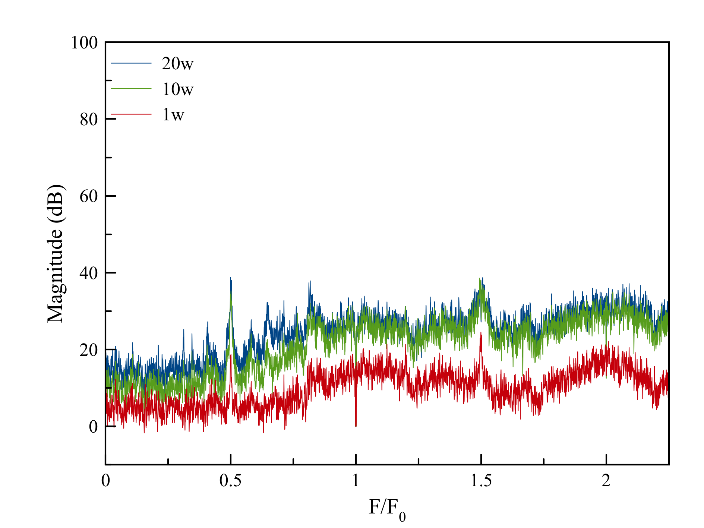
***Corrected full spectra (recording scale 200µV) Corrected restricted spectra (recording scale 100µV)**

*The FFT displays are corrected from 0.5 W reference spectrum*


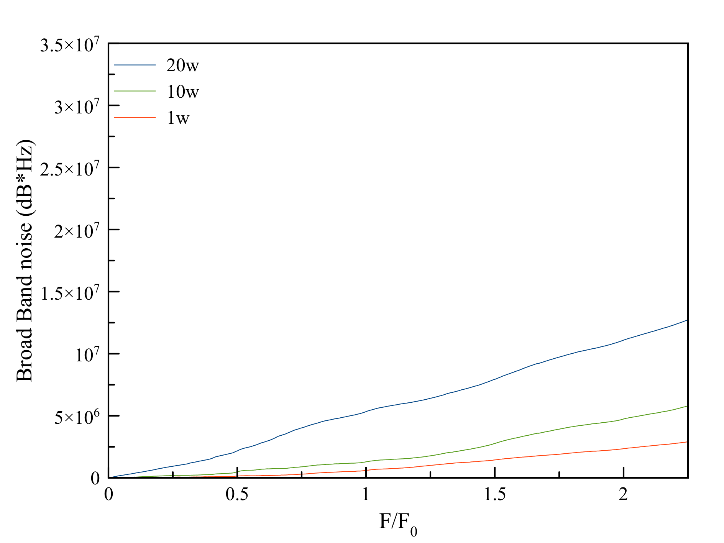

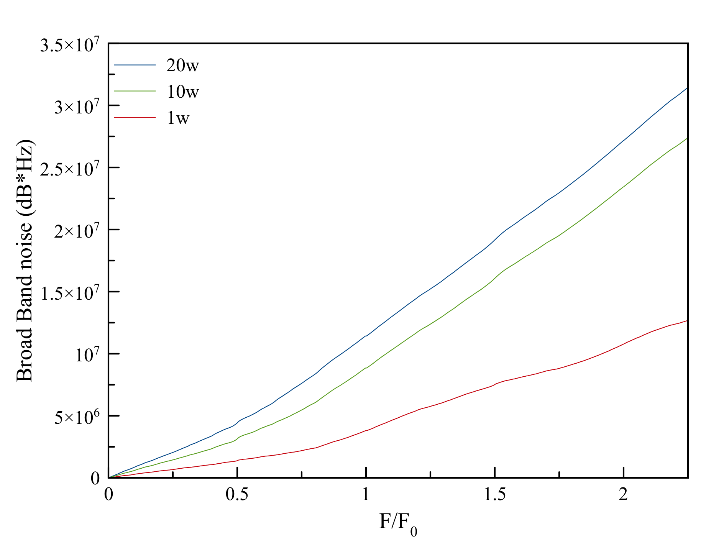
**Full spectra integration(recording scale 200µV) Restricted spectra integration (recording scale 100µV)**

*Integrations made on F/F_0_ = 0 to F/F_0_ = 2.25 on corrected spectra for various transmitted powers*

[SM2]: Comparison between data processing from spectra recorded at full scale range (200µV – left) and restricted scale range (100µV – right)


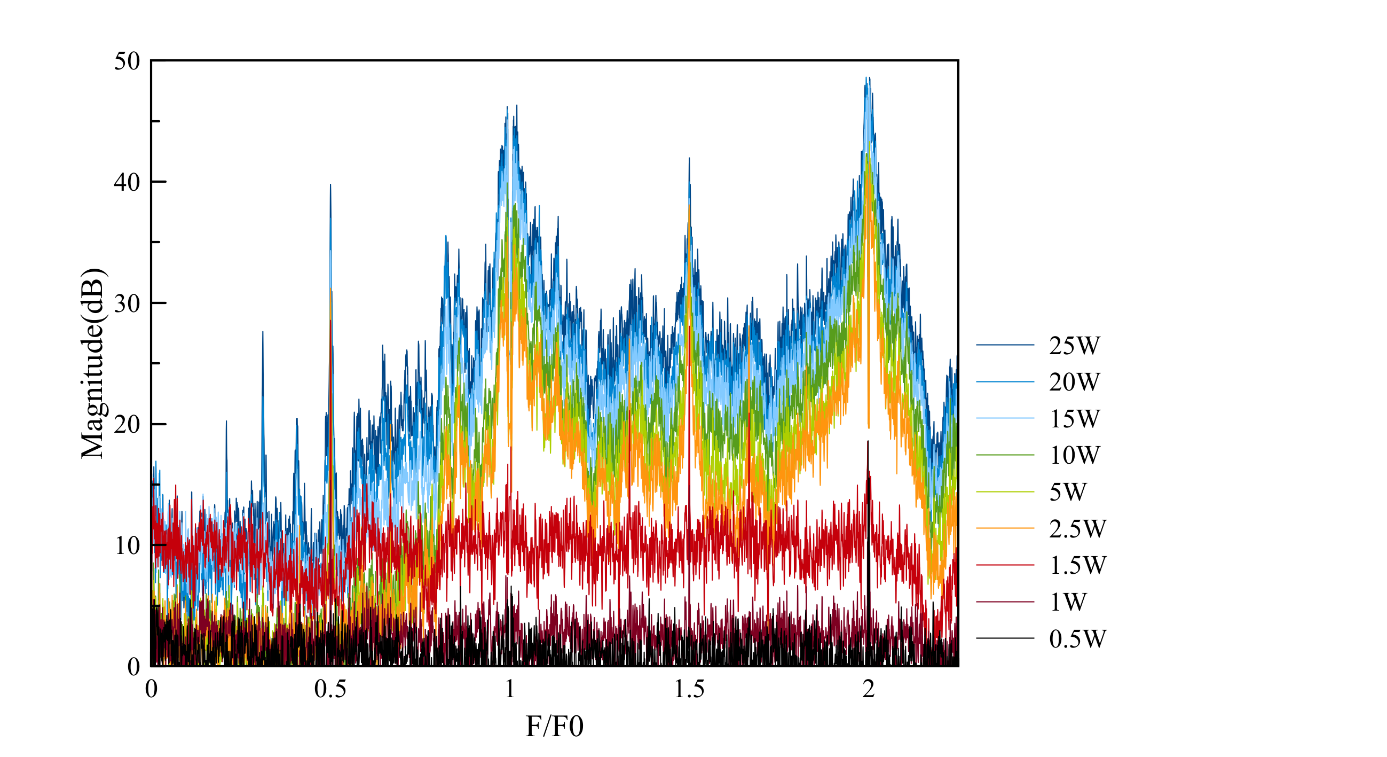


[SM3] : spectra treated by subtracting them at 0.5W


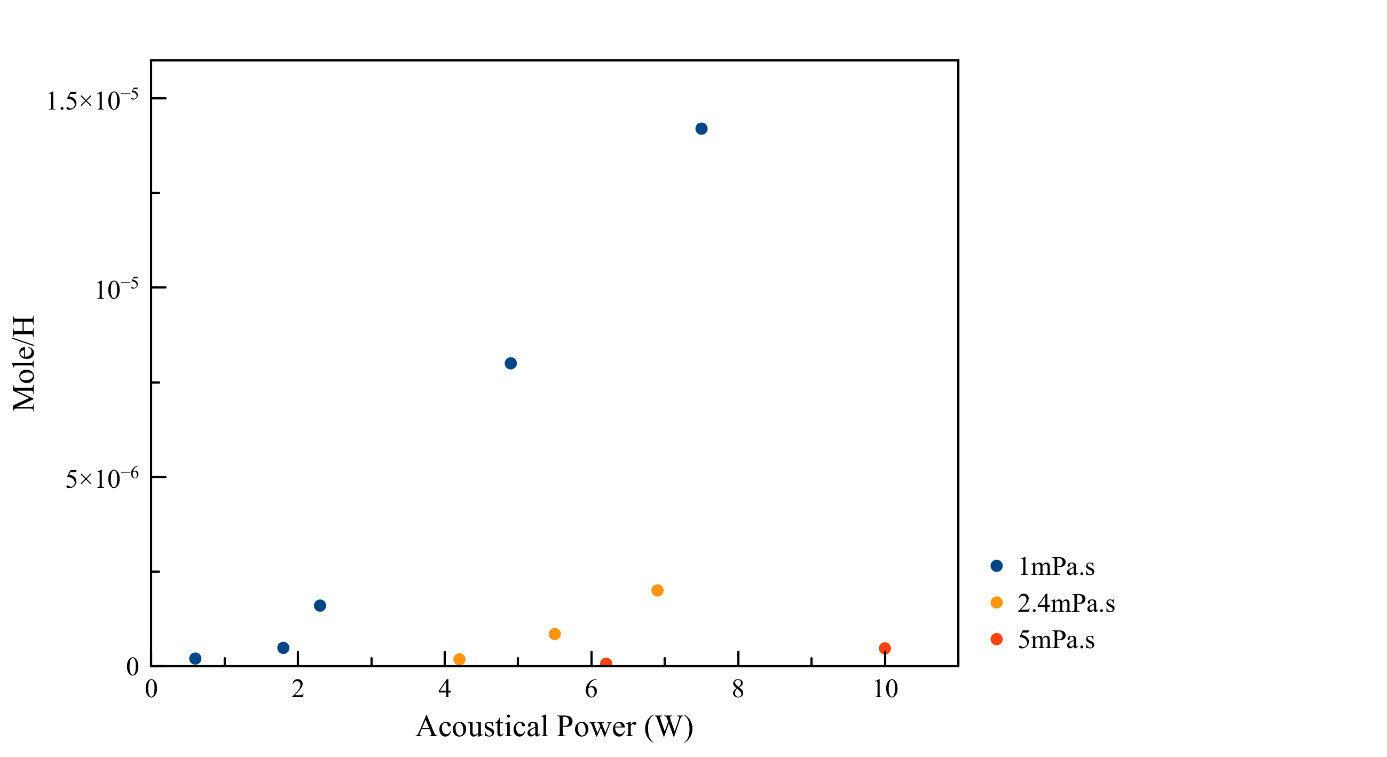


[SM4]: KI dosimetry in molybdate using UViLine 9400C: 350nm

| Viscosity (mPa.s) | Acoustic Power (W) | H_2_O_2_ Production Rate mol/h | Photon number (5ms sampling) |
| --- | --- | --- | --- |
| 1 | 0.6 | 2.00E-07 |  |
| 1 | 1.8 | 4.80E-07 | 13 |
| 1 | 2 |  | 235 |
| 1 | 2.3 | 1.60E-06 |  |
| 1 | 2.5 |  | 272 |
| 1 | 3 |  | 298 |
| 1 | 5 | 8.00E-06 | 741 |
| 1 | 7.5 | 1.40E-05 |  |
| 1 | 10 |  | 3035 |
| 2.4 | 1.8 |  | 8 |
| 2.4 | 2 |  | 998 |
| 2.4 | 2.5 |  | 1275 |
| 2.4 | 4.2 | 1.80E-07 |  |
| 2.4 | 5 |  | 2320 |
| 2.4 | 5.5 | 8.50E-07 |  |
| 2.4 | 6.9 | 2.00E-06 |  |
| 2.4 | 10 |  | 3298 |
| 2.4 | 15 |  | 3110 |
| 2.4 | 20 |  | 3123 |
| 3.2 | 10 |  | 7 |
| 3.2 | 12.5 |  | 835 |
| 3.2 | 15 |  | 814 |
| 3.2 | 20 |  | 2266 |
| 5 | 6.2 | 6.30E-08 |  |
| 5 | 10 | 4.70E-07 |  |
| 15 | 3.8 | 5.40E-07 |  |
| 15 | 4 | 2.50E-08 |  |
| 15 | 10 |  | 113 |
| 15 | 12.5 |  | 231 |
| 15 | 15 |  | 193 |
| 15 | 20 |  | 420 |
| 32 | 10 | 2.70E-07 |  |
| 54 | 9.5 | 4.10E-08 |  |
| 54 | 11 | 2.50E-07 |  |

[SM5]: H_2_O_2_ Production Rate mol/h present in our reactor for different PEG concentrations with corresponding acoustic power as well as photon count.


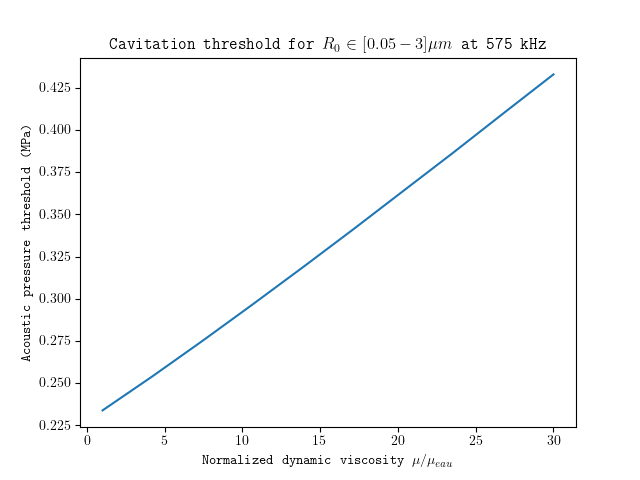


[SM6]: Modeling of cavitation threshold express in Pressure (MPa) according to normalized dynamic viscosity.


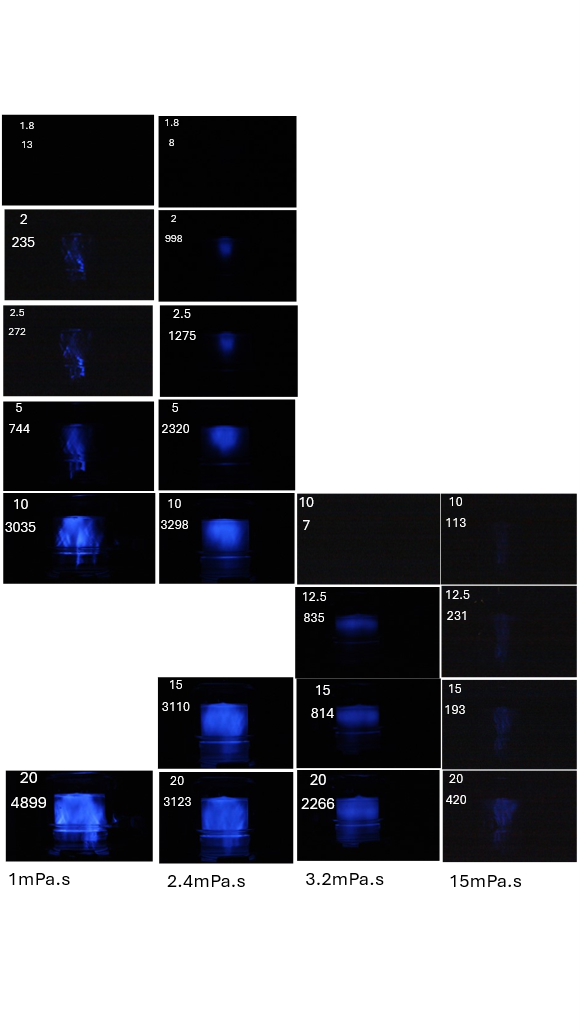


[SM7]: Images show SCL in water and PEG for (on the left side) a viscosity of 2.4mPa.s, 3.2mPa.S, and a viscosity of 15mPa.s (on the right side). Photographs are annotated with the corresponding acoustic power (top) and the number of photons emitted (bottom)


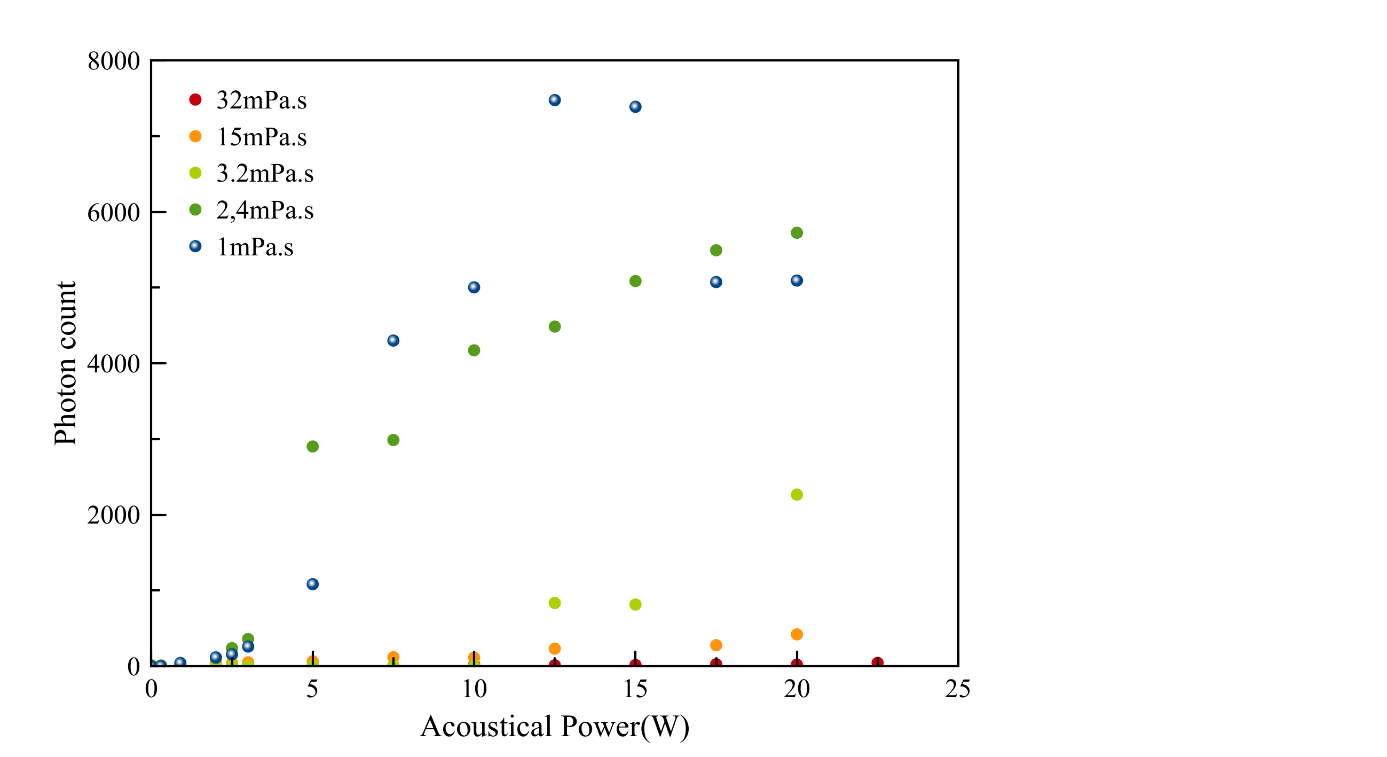


[SM8]: photon count according to acoustic power at various viscosities.
